# Predicting metastasis at initial diagnosis and radiotherapy effectiveness in patients with metastatic osteosarcoma

**DOI:** 10.1007/s00432-023-04869-x

**Published:** 2023-05-24

**Authors:** Wenhao Chen, Xinyu He, Zhiyu Yan, Xiuquan Lin, Guannan Bai

**Affiliations:** 1 Department of Orthopedic Surgery, National Children’s Regional Medical Center, National Clinical Research Center for Child Health, The Children’s Hospital, Zhejiang University School of Medicine, 3333 Binsheng Road, Hangzhou, 310052 Zhejiang China; 2 Department of Child Health Care, National Children’s Regional Medical Center, National Clinical Research Center for Child Health, The Children’s Hospital, Zhejiang University School of Medicine, 3333 Binsheng Road, Hangzhou, 310052 Zhejiang China; 3 Department of Neurology, Massachusetts General Hospital, 55 Fruit Street, Boston, MA 02114 USA; 4 Department for Chronic and Non-Communicable Disease Control and Prevention, Fujian Provincial Center for Disease Control and Prevention, 386 Chong’an Road, Fuzhou, 350012 Fujian China; 5 The School of Public Health, Fujian Medical University, 1 North Xuefu Road, Fuzhou, 350122 Fujian China

**Keywords:** Osteosarcoma, Metastasis, Risk, Radiotherapy, Nomogram, Propensity score matching

## Abstract

Osteosarcoma is a primary malignant bone tumor affecting mostly children and adolescents. The overall 10 year survivals of patients with metastatic osteosarcoma are typically less than 20% in the literature and remain concerning. We aimed to develop a nomogram for predicting the risk of metastasis at initial diagnosis in patients with osteosarcoma and evaluate the effectiveness of radiotherapy in patients with metastatic osteosarcoma. Clinical and demographic data of patients with osteosarcoma were collected from the surveillance, epidemiology, and end results database. We randomly split our analytical sample into the training and validation cohorts, then established and validated a nomogram for predicting the risk of osteosarcoma metastasis at initial diagnosis. The effectiveness of radiotherapy was evaluated by performing propensity score matching in patients underwent surgery + chemotherapy and those underwent surgery + chemotherapy + radiotherapy, among patients with metastatic osteosarcoma. 1439 patients met the inclusion criteria and were included in this study. 343 of 1439 had osteosarcoma metastasis by the time of initial presentation. A nomogram for predicting the likelihood of osteosarcoma metastasis by the time of initial presentation was developed. In both unmatched and matched samples, the radiotherapy group demonstrated a superior survival profile comparing with the non-radiotherapy group. Our study established a novel nomogram to evaluate the risk of osteosarcoma with metastasis, and demonstrated that radiotherapy combined with chemotherapy and surgical resection could improve 10-year survival in patients with metastasis. These findings may guide the clinical decision-making for orthopedic surgeons.

## Introduction

Osteosarcoma is a primary malignant bone tumor affecting mostly children and adolescents (Wang et al. 2020). It has been reported that the 5 year survival of patients with osteosarcoma has increased from less than 20% in 1970s to 60–70% nowadays, thanks to the advancement of surgical resection with adequate surgical margins and neoadjuvant chemoradiotherapy (Miwa et al. 2019). However, approximately 15–20% patients with osteosarcoma are expected to have clinically detectable metastases at the time of initial presentation (Isakoff et al. 2015), with most of the metastases occurring in lungs. The overall 10 year survivals of patients with metastatic osteosarcoma are typically less than 20% in the literature (Kansara et al. 2014) and remain concerning. Moreover, outcomes with current standard therapies for these metastatic osteosarcomas are less favorable (Meyers et al. 2011). While radiotherapy is still one of the most common treatments for osteosarcoma in clinical practice, it is arguable that whether radiotherapy improves the survival of patients with metastatic osteosarcoma (Jafari et al. 2020; Heng et al. 2020; Seidensaal et al. 2021).Therefore, a better understanding of the risk factors and exploration of effective therapeutic approaches for metastatic osteosarcoma at initial diagnosis are pressing.

A few studies exist that to predict the survival of osteosarcoma patients (Wang et al. 2022; Wu and Zhang 2020; Zheng et al. 2018). Nevertheless, a few studies exist that predict the risk of metastasis at initial diagnosis in patients with osteosarcoma. Lu et al. established a nomogram to predict distant metastasis in osteosarcoma (Lu et al. 2021). However, the nomogram required not only the risk factors for metastasis in osteosarcoma patients, but also some treatment-related variables which were not available before therapeutic interventions. More recently, Chen et al. proposed a diagnostic nomogram for predicting distant metastasis in patients with osteosarcoma (Chen et al. 2021a), but the prediction performance is less favorable as indicated by the relatively small area under the receiver-operating characteristic curve (AUC). With respect to the treatment for the patients with metastatic osteosarcoma, radiotherapy is one of the most commonly used methods in clinical practice, besides chemotherapy and surgical resection. However, it is arguable that whether radiotherapy improves survival when treating patients with metastatic osteosarcoma (Jafari et al. 2020; Heng et al. 2020; Seidensaal et al. 2021). These previous studies are also limited by small sample sizes or significant differences in baseline characteristics.

Based on a nation-wide database, here, we aimed to develop a nomogram for predicting the risk of metastasis at initial diagnosis in patients with osteosarcoma. Furthermore, patients with metastatic osteosarcoma at initial diagnosis were categorized into the surgery + chemotherapy group and surgery + chemotherapy + radiotherapy group. We performed the propensity score matching (PSM) analysis to address systematic differences in observed baseline characteristics between the two groups. Finally, 10 year survival of each group was calculated to evaluate the effectiveness of radiotherapy in patients with metastatic osteosarcoma by the time of initial presentation.

## Methods

### Patient selection

Clinical and demographic data of patients with osteosarcoma were collected from the surveillance, epidemiology, and end results (SEER) database (https://seer.cancer.gov). The SEER database is maintained by the US National Cancer Institute and composed of records from 18 cancer registries and covers approximately 30% of the US population. We received acceptance of the data access agreement from the SEER administration. No Institutional Review Board approval was required for this study, since no personal-identifying information is included in our dataset. Patients diagnosed with osteosarcoma were searched in the SEER Research Plus Data with 18 registries, Nov 2020 Sub (2000–2018), under the case-listing session protocol.

### Inclusion and exclusion criteria

Patients were included in our analytical sample based on the following inclusion and exclusion criteria: Patients were diagnosed with osteosarcoma between 2000 and 2018. Patients should be actively followed up. The histologic type based on ICD-O-3 includes “9180: Osteosarcoma, NOS”, “9181: Chondroblastic osteosarcoma”, “9182: Fibroblastic osteosarcoma”, “9183: Telangiectatic osteosarcoma”, “9184: Osteosarcoma in Paget disease of bone”, “9185: Small cell osteosarcoma”, “9186: Central osteosarcoma”, “9187: Intraosseous well differentiated osteosarcoma”, “9192: Parosteal osteosarcoma”, “9193: Periosteal osteosarcoma”, “9194: High grade surface osteosarcoma”, and “9195: intracortical osteosarcoma”. Site and morphology diagnosis were microscopically confirmed using the biopsy specimen at the time of diagnosis. Patients whose reporting source were “Autopsy only” or “Death certification only”, patients who did not undergo surgery treatment, patients whose osteosarcoma was not the only and the first malignancy, and patients who had missing information were excluded from the study.

### Data elements

The demographic and clinical variables included in the study are gender, race, age at initial diagnosis, primary tumor site, histological subtype, and tumor grade, American Joint Committee on Cancer (AJCC) T/N/M, treatment, vital status, and follow-up time. Patients’ ages were categorized into 0–20 and > 20 years old, based on the peak incidence in children and adolescents, and a second peak in the elder people over 50. The race categorization was defined as white, black, American Indian, and Asian and Pacific Islander. Primary tumor sites included axial location and extremities. Histologic subtypes included 9180–9187 and 9192–9194, according to the ICD-O-3 code for osteosarcoma. Tumor grades were categorized into “well differentiated”, “moderately differentiated”, “poorly differentiated”, “undifferentiated”, and “unknown”. All TNM classifications were staged according to the 6th edition AJCC Staging Manual. Finally, only patients with complete information were collected into this study.

### Statistical analyses

R software (Version 3.6.1; R Foundation for Statistical Computing; Vienna, Austria) and SPSS (Version 22.0; IBM Corp. Armonk, NY, USA) were used to perform data analysis for the study.

To identify the risk factor and perform prediction, we randomly split our analytical sample into the training and validation cohorts at a 2:1 ratio. The training cohort was used to establish a nomogram for predicting the risk of osteosarcoma metastasis at initial diagnosis. The least absolute shrinkage and selection operator (LASSO) regression was performed to select the metastasis-related risk factors. The outcome of the model is whether the patient was diagnosed metastasis at initial presentation. All demographic and clinical variables were used as predictors in the model, while only the variables whose coefficients are non-zero with the optimal regularization parameter value (along with their respective coefficients) are used to construct the nomogram. The regularization parameter is optimized using tenfold cross-validation. The validation cohort was used to estimate the predicative accuracy of the nomogram by performing internal validation. The performance of diagnostic prediction model was evaluated with the receiver-operating characteristic (ROC) curves, areas under the curves (AUCs), C-indices, and decision curves. We also used calibration curves to examine the discrepancy between the predicted metastasis and the actual metastasis.

To evaluate the effectiveness of radiotherapy in patients with metastatic osteosarcoma, we identified patients with metastatic osteosarcoma in our analytical sample. The outcomes here were defined as the time from diagnosis to death from overall causes (overall survival, OS) and the time from diagnosis to death attributed to osteosarcoma (cancer-specific survival, CSS). “Surgery + chemotherapy” or “surgery + chemotherapy + radiotherapy” was defined as dichotomous exposures. The baseline clinical and demographic characteristics between the two groups were compared using the Pearson test and standardized mean difference (SMD). PSM was carried out to balance the different characteristics between the two groups. The PSM ratio was set as 1:2, and the caliper was set as 0.05. The Kaplan–Meier method and the log-rank test were performed to compare the 10 year CSS and OS survivals between the two groups, both before and after PSM.

## Results

In total, 4933 patients with osteosarcoma were identified in the SEER database. 3494 patients were excluded from this study according to the exclusion criteria, including 863 cases from 2000 to 2004, 929 cases from 2005 to 2009, 913 cases from 2010 to 2014, and 789 cases from 2015 to 2018. There was no significant difference among these 4 time slots (*P* > 0.05). 1439 patients met the inclusion criteria and were included in this study. The patients were divided into a training cohort (*n* = 959) and a validation cohort (*n* = 480). We observed no significant differences of baseline variables between the two cohorts (*P* > 0.05, Table 1). Among those 1439 patients included in this study, 343 of them had osteosarcoma metastasis by the time of initial presentation.

**Table 1:** Baseline variables between the training and validation cohorts

Variable	Training group 959	Validation group 480	*P* value
Sex			0.115
Male	520	282	
Female	439	198	
Age			0.539
≤ 20	475	229	
> 20	484	251	
Race			0.424
W	745	361	
B	106	66	
AI	10	7	
API	98	46	
T			0.572
T1	392	186	
T2	471	240	
T3	31	22	
T4	65	32	
N			0.446
N0	887	439	
N1	30	13	
N_X_	42	28	
Grade			0.660
G1	37	9	
G2	44	23	
G3	240	142	
G4	342	149	
Gx	296	157	
Primary site			0.461
Trunk	246	125	
Limbs	713	355	

*W* white, *B* black, *AI* American Indian, *API* Asian and Pacific Islander

**Fig. 1:**
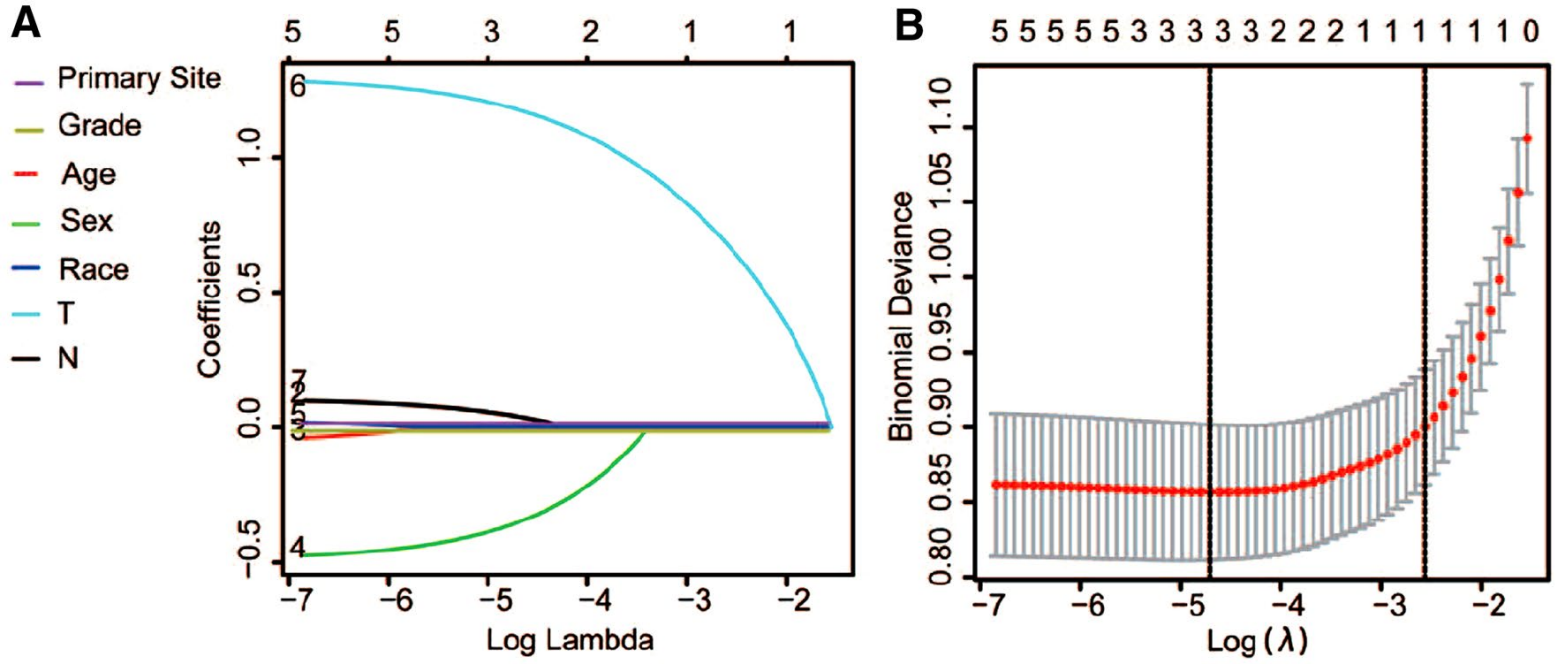
LASSO regression identifying the risk factors of osteosarcoma metastasis at initial diagnosis (**A** Coefficients, Purple for Primary site, Yellow for Grade, Red for Age, Green for Sex, Blue for Race, Light Blue for T, Black for N, and Purple and yellow lines were overlapped with blue line; **B** Binominal Deviance)

**Fig. 2:**
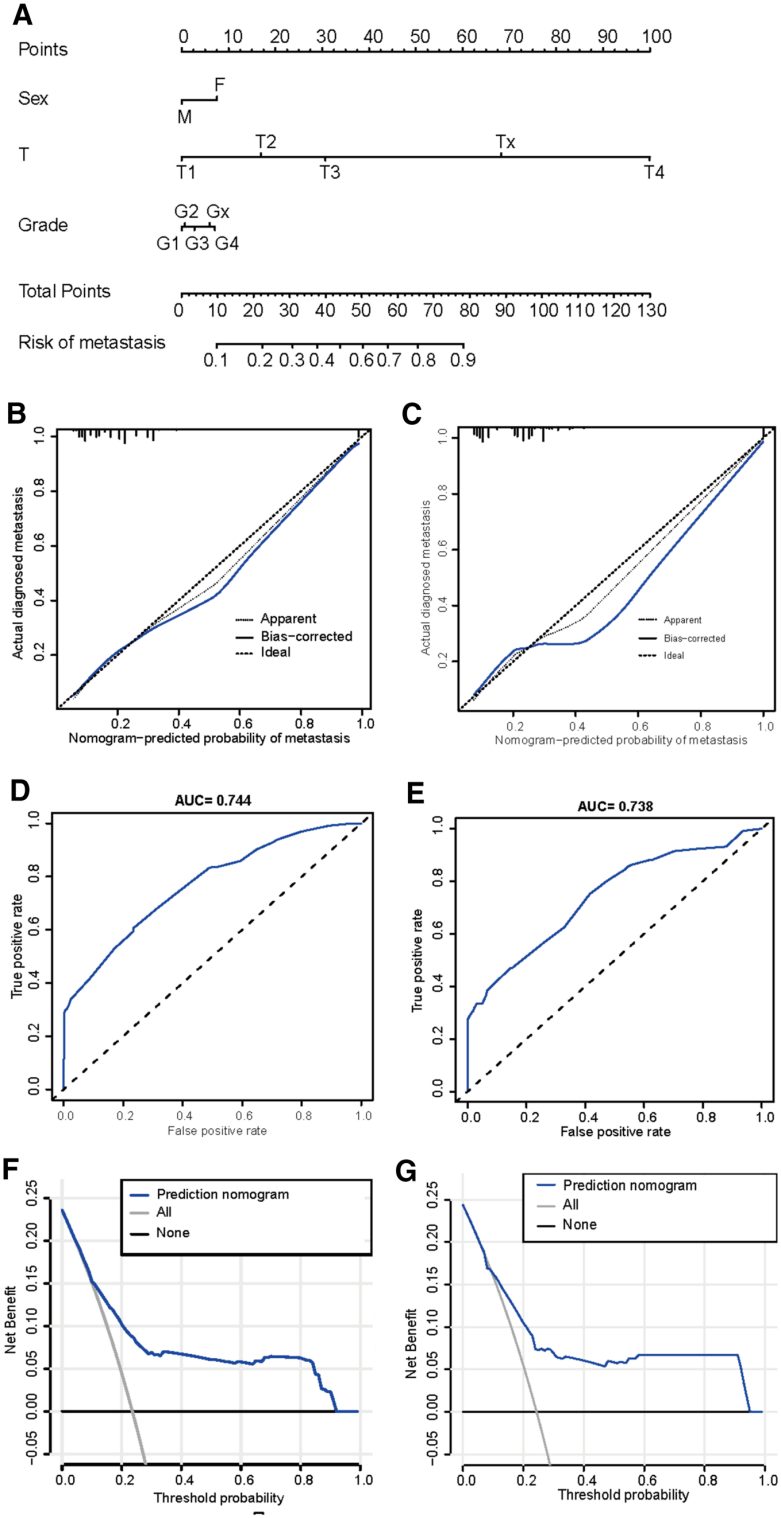
Development, calibration, and evaluation of model predicting osteosarcoma metastasis by the time of initial presentation (**A**: nomogram predicting the likelihood of osteosarcoma metastasis by the time of initial presentation; **B**, **C**: calibration curves for the nomogram with the training and validation cohorts, respectively; **D**, **E**: receiver-operating curves (ROC) for the nomogram, with the training and validation cohorts, respectively; **F**, **G**: decision curves for the nomogram, with the training and validation cohorts, respectively)

The LASSO output demonstrated that sex, tumor stage of T(AJCC, tumor size), and tumor grade were found to be variables with non-zero coefficients when lambda is set to the optimal value based on the cross-validation (Fig. 1). These variables were used to develop the nomogram for predicting the likelihood of osteosarcoma metastasis by the time of initial presentation (Fig. 2A). The C-indices of the nomogram for the training and validation cohort were 0.747 (95% CI 0.704–0.783) and 0.705 (95% CI 0.692–0.734), respectively. The ROC curves of the nomogram-based diagnostic predicting model demonstrated a good predictive accuracy in both training and validation cohorts (Fig. 2B, C). The AUC of the nomogram with respect to the training and validation cohort were 0.744 and 0.738, respectively. The calibration plot of the nomogram showed that the model was well calibrated for both training and validation cohorts (Fig. 2D, E). The decision curves of the nomogram revealed the model had a higher net benefit than categorizing all patients as osteosarcoma metastasis across almost all threshold probabilities (Fig. 2F, G).

In our sample, 343 patients were diagnosed osteosarcoma metastasis by the time of initial presentation, among which 70 patients were treated by surgery and chemotherapy, and 273 patients were treated by surgery + chemotherapy + radiotherapy. Statistically significant differences in the variables including age group (*P* < 0.001), tumor grade (*P* < 0.05), and primary site (*P* < 0.001) were detected between the two treatment groups (Table 2). With PSM balancing these variables, 57 patients in the non-radiotherapy group were matched to 88 patients in the with-radiotherapy group. No significant difference was observed in any variable between the 2 groups after matching (Table 2).

**Table 2:** Characteristics of patients with metastatic osteosarcoma before and after PSM

Variable	Before PSM			*P* value	After PSM			*P* value
	No-radio 70	Radio 273	SMD		No-radio 57	Radio 88	SMD	
Sex				0.438				0.870
Male	37	139	0.039		28	42	0.052	
Female	33	134			29	46		
Age				0.001				0.727
≤ 20	21	155	0.058		20	34	0.038	
> 20	49	118			37	54		
Race				0.593				0.490
W	49	201			39	70		
B	13	34	0.156		10	9	0.134	
AI	1	4	0.003		1	1	0.000	
API	7	34	0.081		7	8	0.116	
T				0.259				0.854
T1	5	50			2	4		
T2	38	134	0.104		31	52	0.070	
T3	8	12	0.219		5	5	0.055	
T4	19	77	0.024		19	27	0.039	
N				0.968				0.750
N0	54	209			43	62		
N1	5	22	0.035		4	9	0.135	
N_*X*_	11	42	0.009		10	17	0.096	
Grade				0.011				0.939
G1	0	9			0	0		
G2	2	13	0.114		2	2	0.105	
G3	15	66	0.066		12	21	0.064	
G4	18	106	0.298		16	26	0.040	
Gx	35	79	0.418		27	39	0.052	
Primary site				0.001				0.859
Trunk	32	59	0.480		36	30	0.087	
Limbs	38	214			21	58		

*W* white, *B* black, *AI* American Indian, *API* Asian and Pacific Islander

**Fig. 3:**
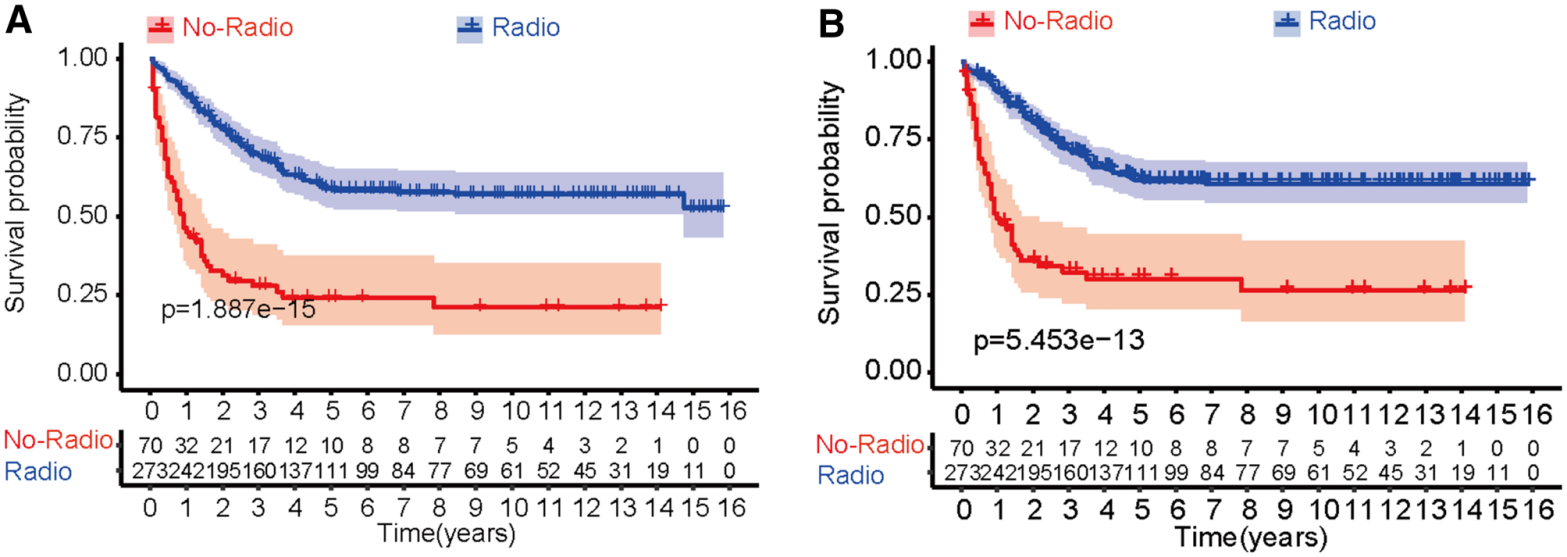
Kaplan–Meier survival curves illustrating metastatic osteosarcoma 10-year overall survival (OS) and cancer-specific survival (CSS), before PSM (**A** 10-year OS, **B** 10-year CSS, no-radio indicates non-radiotherapy group, and radio indicates radiotherapy group; numbers below the graphs: size of risk set at each time point)

**Fig. 4:**
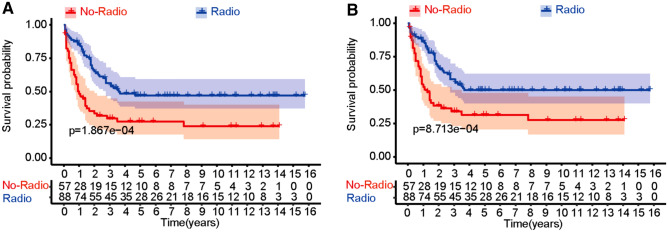
Kaplan–Meier survival curves illustrating metastatic osteosarcoma 10-year OS and CSS, after PSM (**A** 10 year OS, **B** 10-year CSS. No-radio indicates non-radiotherapy group, and radio indicates radiotherapy group)

Before PSM, the unadjusted OS and CSS of the no-radiotherapy group and the with-radiotherapy group were analyzed using the Kaplan–Meier method and log-rank test (Fig. 3). There were significant differences in the 10 year survival probability between the two groups, both for CSS (*P* < 0.001) and OS (*P* < 0.001). After PSM, the difference between the two groups remained significant regarding the CSS (*P* < 0.001) and OS (*P* < 0.001, Fig. 4). In both unmatched and matched samples, the radiotherapy group demonstrated a superior survival profile comparing with the non-radiotherapy group.

## Discussion

Osteosarcoma is the most prevalent form of malignant bone cancer. The malignancy is predominantly derived from the epiphysis of long bones and occurs mainly in children and adolescents (Isakoff et al. 2015; Chen et al. 2021a, 2021b). The osteosarcoma with distant metastasis still results in a poor prognosis (Rickel et al. 2017). Less than 30% of patients with metastatic osteosarcoma can survive with a multimodal treatment combining radiotherapy, chemotherapy, and surgical resection (Kager et al. 2003). Therefore, it is imperative to construct reliable models to predict the risk of metastasis at initial diagnosis for osteosarcoma patients. It is also crucial to assess if radiotherapy is effective in conjunction with chemotherapy and surgical resection in treating metastatic osteosarcoma. In our study, independent risk factors associated with metastasis by the time of initial presentation were identified and used to construct a nomogram. Additionally, the outcomes with two sets of commonly used treatment combinations were investigated.

A nomogram is a powerful and convenient mathematical tool to predict medical outcomes. In this study, we developed a nomogram predicting the risk of metastasis at initial diagnosis in patients with osteosarcoma. By performing the LASSO regression, sex, T-stage tumor, and tumor grade were selected as risk factors for the development of the nomogram. This finding was generally in consistent with previous literatures (Chen et al. 2021a; Miller et al. 2013; Xu et al. 2022), besides these studies have found age to be a risk factor of metastases. Possible reasons for this discrepancy would be the datasets extracted from the SEER database were different between this study (2000–2018) and previous ones, and the strategies in selecting the potential variables were not the same as well. The nomogram demonstrated favorable AUC, C-indices, and decision curves. There was excellent agreement between calibration curves and 45-degree perfect match straight lines. To predict the risk of metastasis at initial diagnosis in a patient with osteosarcoma, an investigator first draws a vertical line from every factor to the “Point” bar in the nomogram and obtains the sum of the points. Then, with the summed points, another vertical line was drawn from the “Total Points” bar to the risk bar to obtain the corresponding probability of metastasis. Previous literature reported several models that predict the risk of osteosarcoma metastasis. Zhang et al. developed a four-gene signature to predict metastasis and prognosis in osteosarcoma (Zhang and He 2021). Similarly, Dong et al. presented a risk score model associated with eight genes for the prediction of osteosarcoma metastasis (Dong and Mao 2019). These models were based on a certain set of genes but not on clinical and demographic characteristics of patients with osteosarcoma, leading to inconvenience in clinical application of the models. Lu et al. (2021) and Chen et al. (2021a) established nomograms based on clinical and demographic variables for predicting distant metastasis in patients with osteosarcoma. However, these two models were limited by variable selection and prediction performance, respectively. To the best of our knowledge, our study is the first to provide a nomogram to accurately evaluate the risk of osteosarcoma with metastasis at the initial diagnosis based on clinical and demographic variables.

Our study also evaluated the effectiveness of surgical resection and chemotherapy, with or without radiotherapy in the context of patients with metastatic osteosarcoma at the initial diagnosis. Before PSM, we analyzed all 343 patients of metastatic osteosarcoma. The unadjusted Kaplan–Meier curve illustrated that the with-radiotherapy group has better CSS and OS than the non-radiotherapy group (both *P* < 0.01). It is noticed that there were significant differences observed in the variables of age (*P* < 0.001), grade (*P* < 0.05), and primary site (*P* < 0.001, Table 2). The non-radiotherapy group included higher percentage of elder patients (> 20 years: 70.0%) than the with-radiotherapy group (43.2%). We also found patients having tumor in the axial location were 45.7% in the non-radiotherapy cohort and only 21.6% in the with-radiotherapy cohort. It has been demonstrated that tumor located in the spine or pelvis leads to poorer prognosis than that located in the extremities (Chen 2019). Moreover, there were more patients with low-grade osteosarcoma in the with-radiotherapy group (G4: 38.8%) than in the non-radiotherapy group (25.7%). Therefore, it cannot be concluded that radiotherapy increases the 10 year survival of patients suffered from metastatic osteosarcoma under these circumstances. To balance observed in baseline characteristics between the two groups, we performed PSM analyses. After PSM, 57 patients in the non-radiotherapy group and 88 in the with-radiotherapy group were matched. As shown in Table 2, the differences in baseline characteristics were dramatically reduced with no statistically significant differences demonstrated *p* > 0.1. We performed the Kaplan–Meier analysis again after PSM and found that there were still statistical differences between the two treatment groups for the 10 year OS and CSS. The treatment effect of radiotherapy in patients of metastatic osteosarcoma was determined without notable bias. Radiotherapy is one of the common therapeutic approaches used in the treatment of malignant tumor, especially in metastatic disease. It works by producing reactive oxygen species, resulting in DNA damage and death of tumor cells (Subash et al. 2012; Marco Durante and Loeffler 2017). Although radiotherapy-related side effects, such as tissue necrosis and damage to central nerve system (especially for axial tumor), need to be taken into consideration (Emma et al. 2015; Anirban Das and Chung 2022), the findings of this study suggest that radiotherapy combined with surgery and chemotherapy can improve 10 year CSS and OS in patients with metastatic osteosarcoma.

Nevertheless, we should acknowledge that our study is not without limitations. First, the number of osteosarcoma patients with metastasis was relatively small. Second, our nomogram was not validated by an external osteosarcoma database, though it has been internally validated by the SEER database. Third, the detailed information about the treatment strategies was not available in the SEER database. Surgical management was defined as surgical resection of the tumor, but the detailed surgical information, such as intra-capsule excision, marginal excision, and wide excision, was not available in the SEER registry. The detailed information about radiotherapy and chemotherapy was also not provided in the SEER registry. These prevented us from investigating different versions of treatments. However, most surgeons from different registries in the United States treated their patients according to the current guidelines.

Our study identified clinical and demographic risk factors of metastasis for osteosarcoma, and established a novel nomogram to evaluate the risk of osteosarcoma with metastasis at initial diagnosis. Furthermore, we demonstrated radiotherapy combined with chemotherapy and surgical resection could improve 10-year CSS and CS with patients with metastasis. These findings may guide the clinical decision-making for orthopedic surgeons.

## Data Availability

De-identified data and statistics are available upon request.
